# Prospective comparison of one year follow-up outcomes for the open complete intrafascial retropubic versus interfascial nerve-sparing radical prostatectomy

**DOI:** 10.1186/2193-1801-3-335

**Published:** 2014-07-01

**Authors:** Wael Y Khoder, Raphaela Waidelich, Alexander Buchner, Armin J Becker, Christian G Stief

**Affiliations:** Department of Urology, University Hospital Munich–Grosshadern, Ludwig-Maximilians-University Munich, Marchioninistrasse 15, 81377 Munich, Germany

**Keywords:** Prostate cancer, Radical prostatectomy, Intrafascial prostatectomy

## Abstract

Current work provides a prospective direct comparison between Open complete intrafascial-radical-prostatectomy (OIF-RP) and interfascial-RP in all outcomes in single centre series. Both techniques were done prospectively in 430 patients. Inclusion criteria for OIF-RP (n=241 patients) were biopsy Gleason-score ≤6 and PSA ≤10 ng/ml while for interfascial-RP (n=189) were Gleason-score ≤7 and PSA ≤15. The perioperative parameters (e.g. operative time, complications etc.), pathologic results, surgical margins and revisions were reviewed. Pre- and postoperative (3 and 12 months) evaluation of continence and potency was performed. All patients have preoperative IIEF-score of ≥15. Continence was classified as complete (no pads), mild (1-2 pads/day) and incontinence (>2 pads/day)**.** Median patients’ age was 63.7 vs. 64.5 years for OIF-RP vs. Interfascial-RP, respectively. Preoperative PSA-level was significantly lower in OIF-RP (5.8 vs. 7.1), otherwise, similar perioperative data in both groups except for more frequent pT3-tumors in interfascial-RP group (18%). No statistical significance regarding continence was observed between OIF-RP vs. Interfascial-RP groups at 3 (82% vs. 85%) and 12 months (98% vs. 96%) postoperatively. Potency rates (IIEF ≥15) after OIF-RP were 96% (≤55 years), 72% (55-65), and 75% (>65 years) at 12 months. The respective rates for interfascial-group were 58%, 61% and 51%. There was an advantage for OIF-RP potency-outcomes without significance over Interfascial-RP in weak potency patients (IIEF=15-18). We conclude that OIF-RP is associated with better functional results without compromising early oncological results compared to interfascial-RP. Complete preservation of periprostatic fasciae provides significantly better postoperative recovery of sexual function even for weak potency patients. Longer follow-up is mandatory to further evaluate the outcome results of this technique.

## Introduction

The primary goal of the management of patients with clinically localized prostate cancer with radical prostatectomy (RP) is the oncologic efficacy followed by preservation of sexual and urinary quality of life. Thus there have been many efforts to improve the technique of radical prostatectomy (Masterson et al. 2011).

With the description the neurovascular bundles (NVB) (Walsh et al. 1983), many surgical techniques became possible to preserve potency. The functional results of RP have not been perfect even in experienced hands. The concomitant good outcomes after alternate forms, e.g. radiotherapy, raised the bar to improve this technique.

Several modifications of the technique were published based on the several published anatomic studies (Stolzenburg et al. 2006a, [b]; Stolzenburg et al. 2007; Walz et al. 2010; Kiyoshima et al. 2004; Costello et al. 2004; Tewari et al. 2003; Menon et al. 2007). Each of which is describing more preservation of the surrounding structures/Fasciae of the prostate. Our participation on the refinement of RP technique, the open complete intrafascial RP "OIF-RP" (Khoder et al. 2011), involves complete preservation of all surrounding fasciae of the prostate. Herein, we present a retrospective cohort analysis of patients prospectively selected for the evaluation of the impact of OIF-RP on the functional outcomes following RP in comparison to the interfascial RP-technique in a single centre series.

## Patients and methods

### Patient population

To evaluate a change in a surgical technique, other factors that influence the surgical outcomes were controlled as follows; the inclusion criteria in this study were clinical stages (cT1-cT2), all surgeries were bilateral nerve sparing, a preoperative International Index of Erectile Function (IIEF-5) score of ≥15 and the availability of a sexual partner. The study design was approved by the committee of our urology department.

430 patients underwent RP between January 2009 and January 2010 in our institute. Patients underwent OIF-RP when biopsy Gleason score ≤6 and PSA ≤10 ng/ml with low tumour size while anatomical interfascial-RP was done in patients with Gleason score ≤ 7 and PSA ≤15. No patient received radiation or hormonal therapy preoperatively or postoperatively. Outcomes in OIF-RP (n = 241 patients) were compared to interfascial-RP (n = 189).

### Surgical techniques

The technique of OIF-RP was previously reported (Khoder et al. 2011). In brief; Endopelvic fascia is not incised and the puboprostatic ligaments are preserved or cut just proximal to prostate with lateralisation landing in a plan between the prostate capsule and levator ani fascia. The prostate capsule is completely bluntly freed laterally from the surrounding fasciae and dorsally from Denonvillier’s fascia maintaining all periprostatic fasciae/nerves intact. Perforator vessels are clipped without coagulation. Dorsal NVB and pre-rectal fat are not actually seen because it lies under anterior layer of Denonvillier’s fascia. The attachment of anterior fascial layer to base of the prostate is separated bluntly at the base of seminal vesicles where there is small fat lobule between both and then cut sharply from the base of the prostate avoiding injury of either prostate capsule or fascia. Ligation and transaction of vasa differentia is left to the end of prostate dissection to provide stability to the prostate and avoids extensive traction on NVB to prevent neuropraxia of NVB and/or capsular/Fascial tears. Any haemostasis is done only with clips and coagulation is completely avoided. Patients with problems in identification of the intrafascial plain or with sticky para-prostatic tissues (post-inflammation or post- biopsy) intraoperatively, where surgeons failed to do OIF-RP, were evaluated as interfascial-RP or excluded from the study as advised by the operating surgeon.

The interfascial-RP technique follows the anatomical rationales (Walz et al. 2010). The endopelvic fascia is opened laterally. The Denonvillier’s fascia is cut laterally to develop the interfascial plain preserving the NVB dorsal to the prostate. The same precautions, like avoiding cauterisation, are followed.

### Outcome measures and statistical analyses

The operative time, transfusion rate, conversions, catheterization time, pathologic results, positive surgical margin rate (PSMs), incidence of complications, and requirement of postoperative interventions were reviewed.

Pre- and postoperative evaluation of continence and potency for all patients was performed by the use of Continence and IIEF-5 questionnaires. Potency and continence data were recorded at 3 and 12 months postoperatively. This was done by a separate hospital unit (third party). Patients not requiring any pads were defined as continent. Requirement for 1–2 pads daily in patients during normal physical activity (e.g. walking) was considered as "mild incontinence" (stress incontinence) and more than 2 pads daily as "incontinence".

Patients achievement of a composite score of 15 points or higher on the IIEF-5 questionnaire, specially responding positively to the following questions: IIEF3-"ability to insert your penis into your partner’s vagina?" and IIEF4-"Did your erections last long enough to have sexual intercourse?", were defined as "potent" regardless if they used a phosphodiesterase type 5 inhibitor (PDE-5 inhibitors) or not. Further, Patients with IIEF-score of 15–19 points were classified as "weak potent" while higher scores as "good potent". Return to baseline was defined as the achievement of the preoperative composite score on the IIEF-score. Patients who did not have return of sexual function at 12 months follow-up were recorded as failures for sexual function outcomes.

Total points of both questionnaires were calculated. Continuous variables were compared between groups using the Mann–Whitney test, and between follow up times (3 and 12 months) using Wilcoxon signed rank test. Categorical variables (e.g. potency and continence outcome, PSM) were tested for significance using the chi-square test. P values below 0.05 were regarded as significant. For all calculations, the software STATISTICA 9 (StatSoft, Tulsa, OK) was used.

## Results

### Operative results

The perioperative patient data of the two groups are summarized in Table 1 (A&B). Median age of total patients in the study population was 63.7 vs. 64.5 years for OIF-RP vs. Interfascial-RP, respectively. Preoperative PSA level was significantly lower in OIF-RP (5.8 vs. 7.1 ng/ml), otherwise, similar perioperative data have been observed in both groups. Weak potency patients were significantly older compared to potent group. There were no relevant postoperative complications necessitating interventions in both study groups. Re-intervention with open surgery never took place.

**Table 1:** **Perioperative patient characteristics for open complete intrafascial and interfascial radical prostatectomy**

**(A)**	OIF-RP median (range) n=38	interfascial median (range) n=26	p value (Mann–Whitney test)
Age [years]	67.1 (49.7 – 74.7)	67.2 (54.4 – 75.2)	0.736
BMI [kg/m^2^]	25.2 (21.4 – 37.7)	26.3 (21.3 – 33.3)	0.173
Initial PSA [ng/ml]	5.8 (0.6 – 9.7)	7.5 (1.7 – 15.0)	0.009
IIEF score before RP	16 (15 – 18)	17 (15 – 18)	0.601
Intraop. blood loss [ml]	100 (100 – 450)	100 (100 – 400)	0.525
Duration of surgery [min]	65 (50 – 75)	65 (45 – 110)	0.942
**(B)**	OIF-RP median (range) n=203	interfascial median (range) n=163	p value (Mann–Whitney test)
Age [years]	62.7 (35.9 – 82.1)	63.5 (41.1 – 77.6)	0.386
BMI [kg/m^2^]	25.6 (17.9 – 33.8)	26.2 (19.4 – 37.5)	0.382
Initial PSA [ng/ml]	5.6 (0.3 – 9.9)	7.0 (0.6 – 15.0)	<0.001
IIEF score before RP	25 (19 – 25)	25 (19 – 25)	0.115
Intraop. blood loss [ml]	100 (50 – 600)	150 (50 – 900)	0.054
Duration of surgery [min]	60 (40 – 120)	65 (45 – 195)	<0.001

A: patients with preoperative weak potency (IIEF score 15 – 18); B: patients with preoperative good potency (IIEF score 19 – 25).

OIF-RP; Open Intrafascial radical Prostatectomy, BMI; Body Mass Index, IIEF; International Index of Erectile Function, intraop.; intraoperative.

Note the change of the total patients’ number due to some missing data or incomplete questionnaires.

### Functional outcomes

Generally, the postoperative functional results (continence and potency) improved significantly in both groups during follow-up from 3 to 12 months. This was for OIF-RP patients as p < 0.001 for both continence and IIEF-score. Corresponding figures in the interfascial-RP group were p = 0.003 for continence and p < 0.001 for IIEF score, respectively.

### Continence

All patients were continent before surgery (0pads/day). Follow-up continence results of each group are demonstrated in Table 2. Patients of OIF-RP group reported to be continent (0–1 pads/day) in 82% of the cases at 3 months, whereas 13% reported minimal stress incontinence (2 pads/day) and 5% required >2 pads/day during the same time period. The respective figures for interfascial-RP were 85%, 12%, and 3% at 3 months. Continence (0–1 pads/day) at 12 months postoperatively was higher with the OIF-RP approach (98% vs. 96%). No statistical significance regarding continence was observed between the two groups at 3 and 12 months postoperatively. Same results without significant difference were observed for both weak and good potency groups as shown in Table 2.

**Table 2:** **Continence outcome after open complete intrafascial and interfascial radical prostatectomy procedures at 3 and 12 months**

**(A)** Follow up	Number of pads/day	OIF-RP percent (n/total)	Interfascial-RP percent (n/total)	p value (chi square test)
At 3 months	0	46 (11/24)	62 (10/16)	0.680
	1	25 (6/24)	19 (3/16)	
	2	25 (6/24)	19 (3/16)	
	>2	4 (1/24)	0 (0/16)	
At 12 months	0	76 (19/25)	78 (14/18)	0.989
	1	20 (5/25)	17 (3/18)	
	2	4 (1/25)	5 (1/18)	
	>2	0 (0/25)	0 (0/18)	
**(B)** Follow up	Number of pads/day	OIF-RP percent (n/total)	Interfascial-RP percent (n/total)	p value (chi square test)
At 3 months	0	66 (101/153)	68 (79/116)	0.915
	1	18 (27/153)	17 (20/116)	
	2	11 (17/153)	11 (13/116)	
	>2	5 (8/153)	4 (4/116)	
At 12 months	0	90 (140/155)	86 (95/110)	0.707
	1	8 (12/155)	10 (11/110)	
	2	1 (1/155)	2 (2/110)	
	>2	1 (2/155)	2 (2/110)	

A: patients with preoperative IIEF score 15 – 18, B: patients with preoperative IIEF score 19 – 25.

Note the change of the total patients’ number due to some missing data or incomplete questionnaires.

### Potency

Preoperative median IIEF-score in the overall cohort was 24 and was equivalent between OIF-RP vs. Interfascial-RP patients (p = 0.148). OIF-RP patients were slightly younger than Interfascial-RP patients as mentioned. There was no significant difference in preoperative sexual frequency between patients in both groups. Of patients who received OIF-RP 46% used a PDF-5 inhibitor on an on demand dosing schedule during the study period. The corresponding value in Interfascial-RP patients was 45% (p = 0.857). overall erectile function sufficient for intercourse (definition in current study: IIEF ≥ 15) with or without the help of PDE-5 inhibitors was reported in 44% and 76% of OIF-RP patients compared to 34% and 56% for Interfascial-RP patients at 3 and 12 months, respectively. Potency results stratified for age are listed in Table 3. The statistical analysis of the potency results revealed significantly improved overall potency in the intrafascial group compared with the interfascial group at 12 months (p < 0.001). An association between patient age and postoperative erectile function is found. Younger men consistently do better in retaining their erections postoperatively across both groups than patients at higher age. Best results were observed in the younger patients (<55 years) with OIF-RP after 12 months (96% IIEF ≥ 15), which was significantly better than the corresponding patients with interfascial-RP (64% IIEF ≥ 15; p = 0.012). Furthermore, significantly better potency was reported by patients >65 years old as compared to Interfascial-RP counterparts at 12 months postoperatively (79% vs. 52% IIEF ≥15; p = 0.006). There were no significant differences between both techniques in the "weak potency" patients although more patients were potent at 12 months after OIF-RP (54%). By further evaluating the postoperative results of the "good potency" group for postoperative IIEF ≥ 19, there was tendency to better results in favour of OIR-RP without significance probably due to the low patient numbers. However, evaluation for IIEF ≥ 15 shows similar results as mentioned before (Table 3).

**Table 3:** **Potency follow-up data after open complete intrafascial and interfascial radical prostatectomy**

**(A)** Follow-up	Age	OIF-RP percent (n/total)	Interfascial-RP percent (n/total)	p value
IIEF 15–19 at 3 months	Overall	23% (5/22)	23% (3/13)	0.981
IIEF 15–19 at 12 months	Overall	54% (13/24)	47% (8/17)	0.654
**(B)** Follow-up	Age	OIF-RP percent (n/total)	Interfascial-RP percent (n/total)	p value
IIEF ≥15 at 3 months	Overall	47% (69/146)	35% (35/100)	0.056
	<55	48% (10/21)	31% (4/13)	0.332
	55-65	49% (36/73)	43% (20/47)	0.469
	>65	44% (23/52)	28% (11/40)	0.099
IIEF ≥15 at 12 months	Overall	80% (125/157)	57% (54/94)	<0.001
	<55	96% (23/24)	64% (7/11)	0.012
	55-65	75% (58/77)	61% (25/41)	0.104
	>65	79% (44/56)	52% (22/42)	0.006
**(C)** Follow-up	Age	OIF-RP percent (n/total)	Interfascial-RP percent (n/total)	p value
IIEF ≥19 at 3 months	Overall	31% (45/146)	29% (29/100)	0.760
	<55	33% (7/21)	31% (4/13)	0.877
	55-65	32% (23/73)	30% (14/47)	0.842
	>65	29% (15/52)	28% (11/40)	0.770
IIEF ≥19 at 12 months	Overall	57% (90/157)	46% (43/94)	0.075
	<55	75% (18/24)	55% (6/11)	0.226
	55-65	58% (45/77)	44% (18/41)	0.132
	>65	48% (27/56)	45% (19/42)	0.770

A: patients with preoperative "weak potency" (IIEF score 15 – 18), B and C: patients with preoperative "good potency" (IIEF score 19 – 25).

OIF-RP; Open Intrafascial radical Prostataectomy, IIEF; International Index of Erectile Function.

Note that differences in total numbers of patients are due to some missing data due to incomplete questionnaires.

### Oncological results

Statistically significant higher incidence of pT3 disease and higher postoperative Gleason scores were observed in the Interfascial group, obviously due to the patient selection criteria in this group (biopsy Gleason score ≤7, initial PSA ≤15). Prostatectomy Gleason score was 6 or less, 7 and 8 in 40.8%, 54.6% and 4.6% of OIF-RP patients, and in 66.4%, 32.7% and 0.9% of Interfascial-RP patients, respectively. PSMs were found in 8.8% vs. 18.1% with pT2 disease (p = 0.009) and in 72.2% vs. 51.5% with pT3 disease (p = 0.151) for OIF-RP vs. Interfascial-RP, respectively. At 12 months, the PSA recurrence-free rates in pT2 cases were 98.1% for OIF-RP and 98.9% for Interfascial-RP group (p = 0.608; Table 4).

**Table 4:** **Histopathological features and oncological follow-up for both groups**

	OIF-RP percent (n/total)	interfascial percent (n/total)	p value (chi square test)
pT2	93% (223/241)	82% (155/189)	<0.001
pT3	7% (18/241)	18% (34/189)	
Gleason score			
6	66.4% (148/223)	40.8% (62/152)	<0.001
7	32.7% (72/223)	54.6% (83/152)	
8-10	0.9% (2/223)	4.6% (7/152)	
R0 (in pT2)	91.2% (196/215)	81.9% (122/149)	0.009
R1 (in pT2)	8.8% (19/215)	18.1% (27/149)	
R0 (in pT3)	27.8% (5/18)	48.5% (16/33)	0.151
R1 (in pT3)	72.2% (13/18)	51.5% (17/33)	
Recurrence-free	98.1% (155/158)	98.9% (93/94)	0.608
After 12 months (in pT2)			

Small variation in case number is due to some missing data.

OIF-RP; Open Intrafascial radical Prostataectomy, R0; free resection margins, R1; positive resection margin.

## Discussion

Recent evidences from the anatomy of the pelvic fascia and NVBs, since its first description (Walsh et al. 1983), has allowed many technical refinements of nerve-sparing techniques during radical proststectomy (Stolzenburg et al. 2007; Walz et al. 2010; Kiyoshima et al. 2004; Costello et al. 2004; Tewari et al. 2003). Although the role of the lateral nerves in continence or erectile function is unclear, the preservation of all tissues between the peri-prostatic fascia and the endopelvic fascia has been proposed as a method to improve functional outcome (Stolzenburg et al. 2010). While the function of these nerves has not been yet clarified, there is a tendency to preserve as much additional nerve fibers as possible to improve functional outcomes of RP (Sievert et al. 2008).

Several modifications of the RP-technique were described as an effort to preserve as many nerve fibers as possible. All reported separately about superior functional results. Montorsi et al. proposed the incision of levator and prostatic fasciae high ventrally at prostate apex (Montorsi et al. 2005). While, Graefen et al. recommended the high incision of periprostatic fascia up to the ventral aspect of the prostate (Graefen et al. 2006.) Savera et al. reported improved potency outcomes after performance of the "Veil of Aphrodite" technique preserving the ventrolateral periprostatic fascia (Menon et al. 2007; Savera et al. 2006). Moreover, Nielsen et al. observed higher potency rates in patients who underwent high anterior incision of the prostatic fascia. Our contribution to the refinement of RP-technique includes preservation of all pelvic fasciae surrounding the prostate as well as puboprostatic ligaments. This results in superior preservation of NVBs and was accompanied with comparable perioperative outcomes compared to the mentioned recent modifications of RP-techniques (Khoder et al. 2011).

The aim of the current work is to provide a direct comparison of patients undergoing an intrafascial procedure with those having an interfascial technique in respect to functional and oncological results in a single high-volume centre experience. The impact of the nerve-sparing procedure was evaluated after controlling all other influencing factors, like surgeon/institutional experience, age of patients and patients’ selection.

The current study showed that OIF-RP was associated with significantly improved sexual function outcomes at 3 and 12 months postoperatively. This confirms the published observations from other high volume centres (Menon et al. 2007; Graefen et al. 2006; Savera et al. 2006; Nielsen et al. 2008; Kaul et al. 2006). The current results are even superior in the younger patient group as compared to later publications. Further, OIF-RP Patients returned to their preoperative baseline function with even higher rates achieving statistical significance between both groups in the age subgroups of patients. This preservation of sexual function associated with intrafascial plane of dissection argues against the concept that the nerves in these fasciae innervate only the corpora cavernosa (Kaul et al. 2006). OIF-RP offered less traction on NVBs which reinforces the concept of improved accuracy in preservation of the neurovascular bundles and prevention of neurapraxia which was also recently suggested to improve the potency outcomes by other surgeons (Nielsen et al. 2008; Chuang et al. 2005; Alemozaffar et al. 2012). Furthermore, current study provided for the first time an evidence that patients with weak potency could profit from OIF-RP more than other nerve sparing surgeries. This should encourage surgeons to advice thorough nerve sparing surgeries for this group of patients.

A significantly improved continence at 3 and 6 months after the intrafascial procedure was reported. (Stolzenburg et al. 2010; Graefen et al. 2006). The authors suggested an advantage of the procedure in early postoperative continence based on the statistical significance in advantage of intrafascial over the interfascial technique postoperatively. Contrary, current study shows no statistical significance regarding continence between the two groups at 3 and 12 months postoperatively. Considering complete continence as the need of 0–1 pads/day (as defined in the mentioned studies), the current continence rate post-OIF-RP will be 98% at 12 months which is higher than the reported incidence after endoscopic, open and laparoscopic intrafascial prostatectomy (93% (Stolzenburg et al. 2010) and 91% vs. 96% (Greco et al. 2010), respectively). Further, a continence rate after "Veil of Aphrodite nerve sparing" and "tension-free" robotic assisted radical prostatectomy of 97% (at 12 months) and 92.4% (at 4 months), respectively was reported. (Kaul et al. 2006; Mattei et al. 2007).

While concerns about OIF-RP regarding improving functional outcomes at the expense of cancer control have been expressed, we believe that the intrafascial plane is less likely to lead to positive surgical margins for pT2 tumours. The current oncological results were not compromised by the OIF-RP for pT2 tumours. The higher incidence of positive surgical margins in Interfascial-RP patients may be explained by the higher Tumour size, PSA level as well as Gleason score in these patients compared to the OIF-RP patients. A very low incidence of detectable PSA in these patients was found 6 to 12 months postoperatively. However, longer follow up is still needed to ascertain biochemical recurrence- free survival in this cohort. Generally, these results are comparable with the published series in literature (Montorsi et al. 2005; Graefen et al. 2006; Savera et al. 2006; Nielsen et al. 2008; Kaul et al. 2006; Chuang et al. 2005; Alemozaffar et al. 2012; Greco et al. 2010; Mattei et al. 2007).

Some important points in the interpretation of the current oncological results remain. Surgical margins in current study were considered as positive even if microscopic and/or over a very small surface area. This should be considered in the interpretation of the results. Further, histological pT3 prostate cancer can be diagnosed only in the presence of attached periprostatic fascia to the prostate specimen. During the OIF-RP procedure, all the periprostatic fasciae are preserved so that this diagnosis is not theoretically possible. However, this observation was already reported for other intrafascial techniques (Stolzenburg et al. 2010; Graefen et al. 2006; Chuang et al. 2005; Mattei et al. 2007) Our pathologists consider T3 disease when there is extracapsular extension rather than just infiltration area. The same observation was reported by (Martinez-Pineiro 2007). Others reported some preserved patches of the fascia in the examined prostate (Stolzenburg et al. 2010), while some reports consider this as a result of excessive peeling of the periprostaic fascia during dissection (Alemozaffar et al. 2012; Secin et al. 2007). Logically, OIF-RP was associated with higher PSMs in pT3 disease (p = 0.151) which encourage adequate preoperative patient selection. Lastly, there was no correlation between PSMs and biochemical recurrence during the first year follow up in current study. This confirms the good oncological results of the presented techniques in spite of the detected PSMs. Longer follow up is mandatory to re-evaluate oncological results of these nerve sparing techniques (Neill et al. 2009).

One limitation of the current study is the non-randomization. A randomised study could have provided more information. A follow-up period of 12 months provides important data but longer follow-up time would provide more solid data about the impact of these techniques on oncological and functional results. Using the same questionnaires and comparison tools to compare both techniques provides solid data despite some possible critical points like use of pad number instead bad weights, other validated questionnaires for evaluation or use of other definitions for continence and potency. There are differences in and in-between both groups, as it should be, albeit, the patients’ number in each group was enough statistically for robust conclusions. Although conclusive, higher patient number may have provided more stability to the statistics in this comparison. Lastly, proper preoperative patients’ selection for OIF-RP is mandatory to avoid high PSMs rate. This selection may be considered to bias current results by some authors. We encourage the concept that the intraoperative suspicion of T3 disease is a clear indication to change for the interfascial technique.

## Conclusion

OIF-RP is associated with better functional results without compromising the oncological results at first year follow-up in comparison to the interfascial-RP. Complete preservation of periprostatic fasciae provides significantly better recovery of postoperative sexual function even in borderline potent men. These results support the inclusion of this modification in the current armamentarium to decrease the morbidity of radical prostatectomy. Longer follow-up is mandatory to evaluate the oncological results of the technique.

### Ethics

Current study has been performed in accordance with the ethical standards. Statistical evaluations were done anonymously and details that might disclose the identity of the subjects under study were omitted. Current manuscript represents a retrospective evaluation and analysis of our prospective and consecutive radical prostatectomy data bank, which was done by a separate hospital unit (third party).
